# Intranasal Nanoemulsions for Direct Nose-to-Brain Delivery of Actives for CNS Disorders

**DOI:** 10.3390/pharmaceutics12121230

**Published:** 2020-12-18

**Authors:** Shiv Bahadur, Dinesh M. Pardhi, Jarkko Rautio, Jessica M. Rosenholm, Kamla Pathak

**Affiliations:** 1 Institute of Pharmaceutical Research, GLA University, Mathura 281406, India; shiv.pharma17@gmail.com; 2 Faculty of Health Sciences, School of Pharmacy, University of Eastern Finland, P.O. Box 1627, FI-70211 Kuopio, Finland; dpardhi9@gmail.com (D.M.P.); jarkko.rautio@uef.fi (J.R.); 3 Pharmaceutical Sciences Laboratory, Faculty of Science and Engineering, Åbo Akademi University, 20520 Turku, Finland; jerosenh@abo.fi; 4 Faculty of Pharmacy, Uttar Pradesh University of Medical Sciences, Saifai, Etawah 206130, India

**Keywords:** nanoemulsions, nose-to-brain delivery, CNS disorders, blood-brain barrier, olfactory pathway

## Abstract

The treatment of various central nervous system (CNS) diseases has been challenging, despite the rapid development of several novel treatment approaches. The blood–brain barrier (BBB) is one of the major issues in the treatment of CNS diseases, having major role in the protection of the brain but simultaneously constituting the main limiting hurdle for drugs targeting the brain. Nasal drug delivery has gained significant interest for brain targeting over the past decades, wherein the drug is directly delivered to the brain by the trigeminal and olfactory pathway. Various novel and promising formulation approaches have been explored for drug targeting to the brain by nasal administration. Nanoemulsions have the potential to avoid problems, including low solubility, poor bioavailability, slow onset of action, and enzymatic degradation. The present review highlights research scenarios of nanoemulsions for nose-to-brain delivery for the management of CNS ailments classified on the basis of brain disorders and further identifies the areas that remain unexplored. The significance of the total dose delivered to the target region, biodistribution studies, and long-term toxicity studies have been identified as the key areas of future research.

## 1. Introduction

Existing therapies have a noteworthy role in the panacea of CNS ailments, resulting in declined fatality rates. However, there are still unresolved issues, and an absolute cure is still elusive for most CNS ailments [1]. The main hindrance is the ability of drugs to cross the blood–brain barrier (BBB) in quantities sufficient to achieve therapeutic levels. It is estimated that the barrier obstructs the permeation of approximately 98% of low molecular weight drugs and of about 100% of macromolecules, leading to a significantly low CNS bioavailability [2]. Consequently, several strategies are being used for the local delivery of active therapeutics to the brain, such as intraparenchymal or intracerebroventricular injections, catheter infusions, mini-pump-assisted intracranial delivery, precise ultrasound methods, or electromagnetic force-field techniques. Nevertheless, these methodologies are invasive, risky, and induce neurotoxic effects at the delivery site, and many of them are not appropriate for chronic treatments.

Thus, the presence of the BBB constitutes the primary limiting factor for drug delivery to the brain through systemic circulation [1]. Several methods are under process to bypass the BBB, and among these, one significant approach is nose-to-brain delivery. The intranasal route is a promising substitute to the above-listed invasive methods for brain drug delivery, as the nasal cavity provides a highly vascularized large absorption surface area for drug administration. This route permits circumvention of the BBB, thereby providing rapid and direct delivery to the brain [3]. Bypassing the BBB, the administered drug reaches the brain, thereby prolonging its residence time within the active site. This route also restricts the unnecessary systemic exposure of drugs and reduces systemic toxicity [4]. The olfactory region of the nasal cavity extends to the cranial cavity and can provide direct access to the brain. Different factors and pharmacokinetic parameters affect the rate of drug transport that are required to be evaluated at the clinical level [5,6]. Presently, nanotechnology-based delivery systems are being given much emphasis for intranasal drug delivery to the brain. Nano-sized drug delivery systems have been extensively studied during the past few decades as a new strategy for circumventing the poor bioavailability of various pharmaceutical drugs [7]. Successful intranasal drug delivery to the brain through nanoemulsions, nanoparticles, liposomes, microspheres, dendrimers, carbon based nanoformulations, and others have been documented in the literature [8].

For example, the intranasal administration of talinolol in nanoemulsions showed higher brain concentrations in rats than with intravenous infusion after 15 min [9]. Various nanoemulsions have been commercialized for biological and medical applications [10].

**Figure 1:**
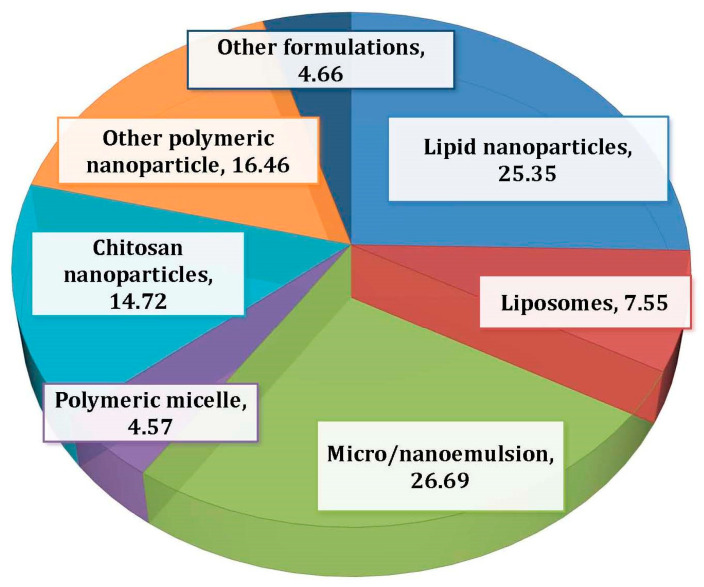
Papers published on nanoemulsion-based nose-to-brain drug delivery systems. The search engine used was Google Scholar. A total of 159 papers were found. Other drug delivery systems included liposomes, transferosomes, ethosomes, metal nanoparticles, etc.

During an extensive literature search from 2015 to January 2020, we found that 159 articles were published on intranasal drug delivery (Figure 1). It was observed that 26% of the reported research was on the nose-to-brain delivery via micro-/nanoemulsions. The present review therefore focuses on the descriptive aspects on nanoemulsion-based nose-to-brain drug delivery. Furthermore, it compiles the research reports on classified CNS disorders and presents the gaps that need to be addressed in the said research arena.

## 2. Pathways for Brain Delivery through the Intranasal Route

The understanding of anatomy and physiology of the nasal cavity is very important for the success of nasal drug delivery systems. The nasal cavity can be divided into three areas: vestibule, respiratory region, and olfactory region. The vestibule region has a small surface area, and drug absorption is insignificant through this region. The respiratory region, on the other hand, is rich in blood capillaries, and thus, it can provide systemic drug absorption and, subsequently, indirect drug delivery to the brain after intranasal administration [11,12]. Trigeminal nerves that are located in the respiratory region can also provide a direct pathway for drugs to the brain [13]. It has been, for example, observed that the respiratory region is most appropriate for the delivery of vaccines through intranasal administration. The olfactory region also plays an important role in the direct drug delivery to the brain and to the cerebrospinal fluid (CSF). Notably, the olfactory region is situated in the upper part of the nasal cavity, which may limit drugs reaching this permeation area [1,14].

**Figure 2:**
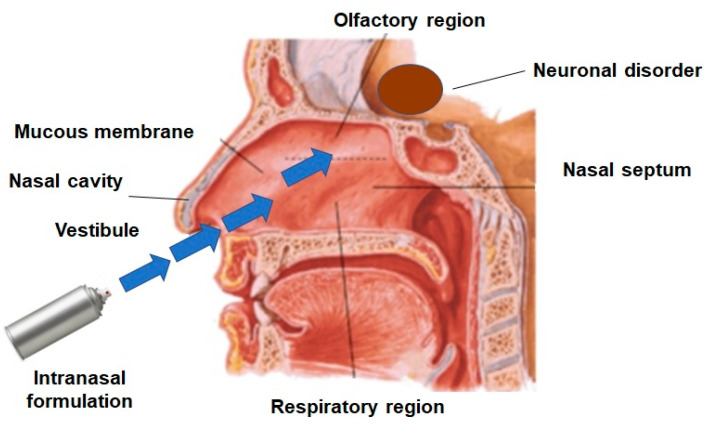
Representative figure on the route of drug transport for nose-to-brain delivery.

The major aim of these drug delivery pathways is to deliver the desired drug concentrations to the site of action. Additionally, the degradation of drugs through metabolism can be diminished, and physical clearance can also be minimized; the overview of drug transport is illustrated in Figure 2 [15]. Highly permeable nasal epithelium allows rapid drug absorption to the brain due to a high total blood flow, porous endothelial membrane, large surface area, and avoidance of first-pass metabolism. The intranasal method can deliver a wide variety of therapeutic agents (small molecules and macromolecules) to the CNS. Several drugs have been shown to be more effective in the CNS when given nasally and provide their therapeutic effects in smaller doses. Further, nasal drug delivery neither requires any modification of the therapeutic agent nor requires the drug to be coupled to any carrier. Nasal drug delivery consisting of various pathways has always been a key development area for both pharmaceutical and medical device companies, presenting compelling advantages over other drug delivery methods [16,17]. The pathways for nose-to-brain delivery have been elaborated on below.

### 2.1. Olfactory Pathway

The olfactory region that is situated in the roof of the nasal cavity is extensively recognized as a possible nose-to-brain drug delivery route for the treatment of various CNS diseases [15]. Drugs can cross olfactory epithelial cells by moving slowly through the tight interstitial space of cells by passive diffusion or across the cell membrane by endocytosis, or they can be transported by neurons [4,18]. Most drugs deposited on the olfactory region are transported extracellularly between cells. Several experimental in-vitro and in-vivo results indicated the clinical relevance of P-glycoprotein in intranasal drug administration. Further, in-vitro experiments have been performed for the testing of drug penetration efflux transporter substrates at the nasal barrier in RPMI 2650 cells and in 3D MucilAir™ nasal models [19].

Olfactory neurons have an important role in drug targeting to the brain by intranasal administration [20]. Drugs are transported into the olfactory bulb through the intracellular axonal channel and subsequently distributed into the brain [21]. The diameter of the olfactory axon in humans is about 0.1–0.7 μm, which indicates that molecules or nanoparticles having diameters within this range can be readily delivered through this pathway [8]. Drug transport through the epithelial pathway is faster than axonal transport. Drug transport through olfactory pathways occurs through extracellular and intracellular mechanisms. Most of the lipophilic drugs are transported by passive diffusion, while hydrophilic drugs are transported by the paracellular route. The molecular weight and lipophilicity of drugs have a significant effect on the drug absorption mechanism. The transcellular mechanism is most relevant to drugs with a very high lipophilicity [4].

### 2.2. Trigeminal Pathway

The trigeminal pathway for nose-to-brain drug delivery has been less explored. The main function of the trigeminal nerve is to pass chemosensory and thermosensory information to the nasal, oral, and ocular mucosa [22,23]. The trigeminal nerve innervates the dorsal nasal mucosa, which reaches the frontal brain and olfactory bulb [24]. Thus, the trigeminal nerve pathway can be one of the potential sites for drug delivery to the brain from the nasal cavity. For instance, a solution of insulin-like growth factor 1 was delivered to brain through the trigeminal and olfactory pathways [25].

### 2.3. Lymphatic Pathway

Drugs can be transported by several extracellular pathways, such as perivascular, perineural, and lymphatic channels from the submucosal area of the olfactory region. These extracellular pathways are linked to olfactory nerves arising from the lamina propria into the olfactory bulb of the brain [8,26]. Therefore, the lymphatic pathway also has a significant role in nose-to-brain drug delivery.

### 2.4. Systemic Pathway

The systemic pathway is an indirect transport system from the nose to brain, and it can be a promising approach for lipophilic drugs with low molecular weights [27,28]. Drugs are then absorbed by the vascular regions of the epithelium membrane of the nasal mucosa and lymphatic system and are further transported to the systemic circulation, thus avoiding the first-pass metabolism [27,29].

## 3. Nanoemulsions

**Figure 3:**
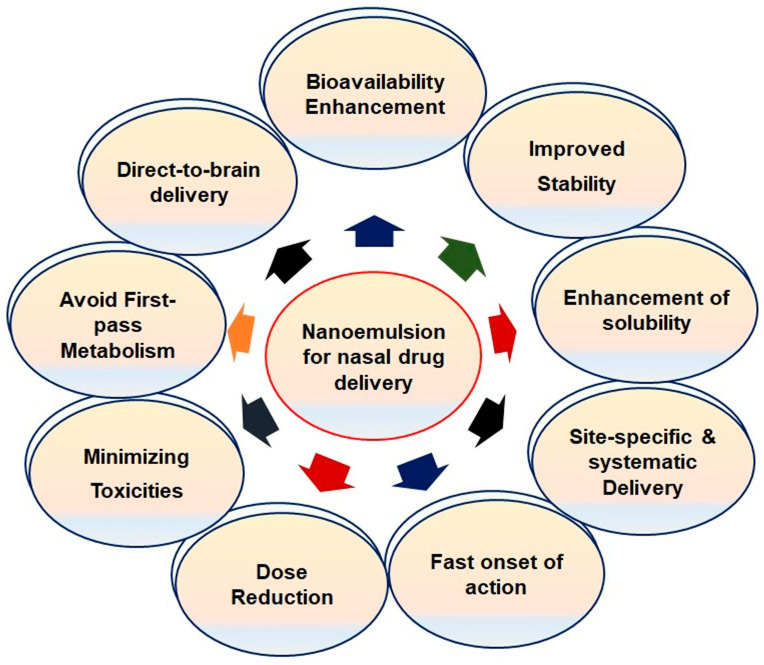
Significant features of nanoemulsions for nasal administration.

Nanoemulsions (NEs) are lipophilic systems with nanoscale globules that can be absorbed through the nasal mucosa. These can be either oil-in-water (o/w) or water-in-oil (w/o) emulsions. Especially, o/w NEs are a promising option for the encapsulation of lipophilic drugs, protecting them from enzymatic degradation, increasing their solubility in liquid media, modulating their drug release, and improving their bioavailability [30]. NEs may be modified to mucoadhesive systems to increase the residence time of the formulation and to overcome the nasal clearance to achieve enhanced mucosal absorption. NEs have also been proven to mitigate the side effects and toxicity of drugs. Several NE formulations, primarily of the o/w type, have been developed for nose-to-brain delivery [31]. Some significant parameters of NEs for intranasal administration are presented in Figure 3. NEs may be developed through different methods by using oil, surfactants, cosurfactants, and water, all of which play a significant role in the permeation of drugs through the nasal mucosa.

### 3.1. Overview of Nanoemulsion Components

#### 3.1.1. Oils

The major problem of new molecular entities in the drug discovery and development pipeline is their poor water solubility, which affects several key properties of therapeutic agents, such as the pharmacokinetic and pharmacodynamic parameters. Hence, oils are used for the development of NE to achieve the maximum solubility of drugs. The lipophilicity of oils is directly proportional to the solubility of drugs [32]. The solubilizing capacity of oils decreases in the order of vegetable oils > medium-chain triglycerides > medium-chain mono- and diglycerides [33]. Further, the solubility of drugs also depends on the concentration of oils in the NE formulations. The globule size of NE increases with an increase in the oil concentration [34]. At the same time, a larger globule size reduces drug permeation from the nasal mucosa. Some oils have permeation-enhancing properties as well. NEs showed selectivity in the uptake of some drugs, such as linolenic acid, polyunsaturated and omega-6 fatty acids, and pinolenic acid [35]. Edmond et al. proved that oleic acid with one *cis*-double bond did not get transported across the BBB, while linoleic acid with two *cis*-double bonds and 18 monocarboxylic acids efficiently entered the brain after intranasal administration [36].

The maximum solubility of a drug can be achieved by striking a correct balance between the concentration of the emulsifying agent (surfactant) and oils. Here, one needs to select the region having maximum emulsification in the phase diagram [34]. Better drug permeation can be achieved through a minimum globule size; hence, formulations having higher globule sizes have less permeation through the nasal mucosa. Several oils have significant permeation-enhancing properties through the nasal mucosa. For example, NE of quetiapine fumarate containing butter oil showed significant nose-to-brain delivery [37]. It was deduced that polar lipids from butter oil enhanced the permeation through the nasal mucosa.

#### 3.1.2. Surfactants

Surfactants are essential components of NE formulation and signify an important role in the surface tension reduction. Surfactants stabilize NE by preventing the phase separation and coalescence of globules. Further, surfactants affect solubilization of the drugs and increase the permeation of drugs through the nasal mucosa due to alterations of the fluidity and damage to the tight junctions of epithelial layers [38]. Several studies report that the globule size of NEs decreases with increasing the concentrations of the surfactants. The lower the globule size, the better the permeation and, hence, the drug concentration in the brain. Nevertheless, the structural integrity of the nasal mucosa is critically affected by surfactants. Hence, the surfactant concentration should be selected carefully, keeping in view the safety considerations of the nasal mucosa [39,40].

#### 3.1.3. Cosurfactants

Surfactants alone are not able to reduce the surface tension to the desired level, because most of the surfactants used in the development of NEs are single-chain surfactants. Therefore, cosurfactants are incorporated to achieve the desired hydrophilic-lipophilic balance (HLB) [41,42]. Cosurfactants increase the fluidity of the formulations by reducing the interfacial tension, which can facilitate emulsification and stabilize the NE. For the development of stable NE, a judicious combination of surfactant and cosurfactant is crucial. The construction of ternary-phase diagrams is a commonly used methodology to optimize the working range and the optimum concentration(s) of oil, surfactant, and cosurfactant. An increase in the concentration of the cosurfactant deceases the globule size of the NE, and, ultimately, the drug concentration will be enhanced. Some most commonly used cosurfactants in the development of NEs for intranasal administration are transcutol-P, butan-1-ol, chiral alcohols, sorbitol, and polyethylene glycol [33].

### 3.2. Significant Factors of Nanoemulsions for Nose-to-Brain Delivery

Several research reports have shown evidence for better drug permeation to the brain from the nasal mucosa by NEs than after conventional oral drug delivery. Apart from the drug permeation-enhancing properties of the surfactants and cosurfactants, NEs have several significant features tailored for brain targeting [10]. Some major features of the NEs are outlined below.

#### 3.2.1. Globule Size

The globule size of the NE plays a very significant role in drug permeation through the nasal cavity. As stated earlier, olfactory and trigeminal transport routes are the major channels for drug delivery to the brain by nasal drug delivery. The average diameter of an olfactory axon is approximately 200 nm in different preclinical species, but in humans, it ranges between 100 to 700 nm [43]. Hence, the globule size of novel formulations should be below 200 nm for successful drug permeation. Ahmad et al. reported that NEs with an average globule size of 100 nm exhibited a higher rate and extent of drug absorption than the average globule size of 700 nm through the olfactory pathway [43]. In addition, the globule size of the NE also affects the retention time of the formulations on the nasal mucosa. Smaller globule size formulations have longer retention times than the larger globule NEs that can be easily removed by nasal clearance, thus having reduced drug absorption [44]. For example, while NEs with average globule sizes larger than 200 nm may exhibit retention times up to 4 h after intranasal administration, NEs having globule sizes of 80 and 200 nm have shown retention times of 16 and 12 h, respectively. Hence, globule sizes of nanoemulsions play a significant role for drug delivery to the brain through intranasal administration [45].

#### 3.2.2. Zeta Potential

The colloidal stability of NE is connected to the zeta potential of the developed formulations. For any colloidal system, zeta potential values exceeding ±30 mV provide electrostatically stabilized systems [46]. Moreover, several reports have shown that the zeta potential has a significant role in the drug retention time of the NE formulations. Mucin found in the nasal mucosa bears a negative charge; hence, formulations carrying positive charges depict good attachment to the nasal mucosa [25]. Several studies have shown that, usually, most of the nasal NEs developed for brain delivery bear negative charges. The values of zeta potential higher than −10 mV of the emulsion indicate the instability of NEs. Therefore, zeta potential is also an important consideration in the development of NEs for nose-to-brain delivery [40].

## 4. Intranasal NEs for Brain Disorders

Even though the nose-to-brain pathway is a proven direct drug delivery approach to the brain, the absorption efficacy of NEs through this pathway is still questionable and awaiting further validation with proof. In one study, the nose-to-brain transport of NEs was traced by the fluorescence bioimaging technique. The localization of NEs in the biological tissues was based on the on-off switching of signals of environment-sensitive embedded dyes (P2 and P4) and two probes (coumarin-6 and DiR) to represent the cargoes. NEs translocation in rats was established by either through an in vitro histological analysis or live imaging. The results evidenced that ≈100 nm globule-sized NEs, decorated or nondecorated with chitosan, had long retention times in rat nostrils and slower mucociliary clearance than larger ones. The P2 signals were traced in the mucosa and trigeminal nerves for all globule-sized groups, while weak P2 signals were identified for chitosan-decorated NEs of 100-nm size in the olfactory bulb. Confocal laser scanning microscopy confirmed the active transport of integral globules in the nasal mucosa and along the trigeminal nerve as attenuated signals. The low intensity of the P4 probe, also representing integral NEs, was identified in the olfactory bulb, and very few signals were detected in the brain. The study demonstrated that NEs as large as 900 nm could not be delivered to the olfactory bulb. However, the coumarin-6 or DiR signals were found in significant quantity along the nose-to-brain pathway that finally reached the brain. Thus, it was evidenced that integral NEs can be delivered to the olfactory bulb, but few were transported to the brain [45]. Therefore, the cargoes should permeate into the brain in greater amounts, proving intranasal administration as a promising drug delivery strategy. Several intranasal NEs have been studied for the treatment of CNS ailments such as Alzheimer’s disease, epilepsy, Parkinson’s disease, migraines, depression, brain tumors, and other related disorders. Some examples of intranasal NEs with their potential outcomes for brain disorders have been compiled in Table 1.

### 4.1. NEs for Alzheimer’s Disease

Many characteristics of the BBB are affected in Alzheimer disease (AD), and these changes, in turn, have implications for the onset, progression, control, and treatment of the disease. In such circumstances, the BBB itself becomes a therapeutic target, and at the same time, it also acts as a formidable barrier against the delivery of drugs to the brain in the treatment of AD. Drugs like acetylcholinesterase inhibitors (galantamine, rivastigmine, and donepezil) and memantine used for AD treatment show poor brain delivery due to unfavorable pharmacokinetics and pharmacodynamics of drugs [59,60].

NEs have garnered considerable interest in research for AD therapeutics due to their attractive features. Sood et al. reported the intranasal delivery of curcumin–donepezil NE for brain targeting in AD. The NE was formulated using Capmul MCM as the oily phase, Cremophor EL and Tween 80 as surfactants, and PEG 400 as a cosurfactant to result in particle sizes less than 50 nm, which are regarded appropriate for intranasal administration. In vivo pharmacokinetic studies in streptozotocin-induced AD model rats revealed higher drug localization in the brain via the intranasal route compared to the intravenous route. The drug clearance from the nasal cavity was also slower after intranasal administration. The pharmacodynamic study of behavioral tasks in rats showed improved memory and learning in the test group treated with NEs compared to the drug alone. A biochemical assessment revealed that acetylcholine levels in the brain were significantly improved in the NE-treated group. The oxidative stress was much lower in animals treated with a combination therapy. Thus, the strategy of the intranasal delivery of an acetylcholinesterase inhibitor with a neuroprotective and anti-amyloid drug appeared to be a promising strategy for the management of AD. The same research group reported the optimization of mucoadhesive NE of curcumin using the Box-Behnken design, which resulted in a high permeation of curcumin across the nasal mucosal layer and was devoid of cytotoxicity in the SK-N-SH cell line [33,61].

Huperzine A (HupA), a reversible acetylcholinesterase inhibitor, is neuroprotective and enhances memory in behavioral animal models [62]. The commercially available oral and injectable formulations of HupA lack brain selectivity [63]. Hence, a new drug delivery system is required to improve the transport and distribution of the drug to the brain. Jiang et al. aimed to develop HupA NE (HupA-NE) for intranasal administration and imprsove its targeting efficiency by modifying HupA-NE with lactoferrin. The optimized HupA-NE with a globule size of 15.24 ± 0.67 nm, polydispersity index of 0.128 ± 0.025, and a zeta potential of −4.48 ± 0.97 mV was modified with lactoferrin. The lactoferrin-HupA-NE was readily taken up into hCMEC/D3 cells (in vitro model for BBB containing P-glycoprotein, multidrug resistance associated protein 1 transporters, and the breast cancer resistance protein) to a greater extent than drug NE without lactoferrin [64,65]. The mechanisms proposed for higher transport to the brain were identified as transcytosis and uptaken by specific transporters. The intranasal administration of lactoferrin-HupA-NE in rats significantly (*p* < 0.05) enhanced drug delivery to the brain compared to HupA-NE. The direct targeting index of lactoferrin-HupA-NE (3.21 ± 0.75) demonstrated brain targeting, and the area under the curve (AUC)_0–α_ for lactoferrin-HupA-NE was significantly higher (*p* < 0.05) compared to HupA-NE [66].

### 4.2. NE for Parkinson’s Disease

**Figure 4:**
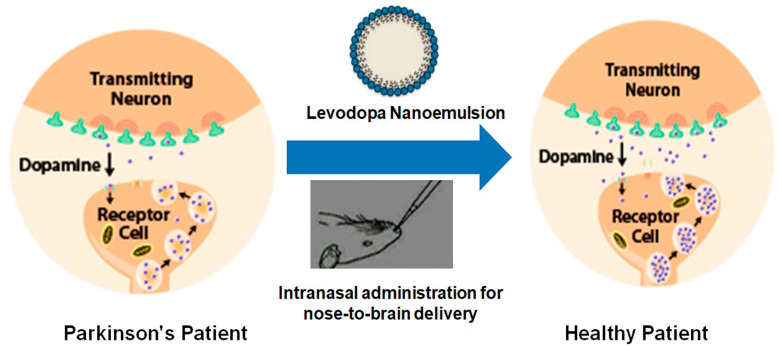
Diagrammatic representation of the effects of drug-loaded nanoemulsion on the dopamine levels in the brain in Parkinson’s disease.

In spite of several developments in drug delivery systems, the treatment of neuronal disorders such as Parkinson’s disease (PD) are still lacking, with limited treatment strategies [67]. Evidence that dopamine loss is the key pathological feature of PD and the subsequent introduction of levodopa revolutionized the treatment of PD (Figure 4). Levodopa is a metabolic precursor of dopamine and is capable of traversing the BBB, where it gets converted into dopamine [68].

Zainol et al. successfully developed a levodopa NE based on palm oil that has high thermodynamic and oxidative stability. Response surface methodology (RSM) was used for the investigation of the influence of the emulsion composition: a mixture of palm and medium-chain triglyceride oil (6–12% *w*/*w*), lecithin (1–3% *w*/*w*), and Cremophor EL (0.5–1.5% *w*/*w*). The researchers compared the effects of the interactions and significant factors associated with the composition of lecithin and Cremophor EL based on the RSM. The authors concluded that the RSM as a beneficial tool for carrying out the optimization study of levodopa NE formulations, and the stability of the levodopa-loaded NE, were correlated to the stabilizing effects of lecithin and Cremophor EL [69].

The intranasal administration of an antioxidant vitamin E loaded with naringenin for the direct brain delivery for PD therapeutics was explored [70]. The characteristics of optimized NE were a droplet size of 38.70 ± 3.11 nm and narrow polydispersity index of 0.14 ± 0.0024. Behavioral studies in Wistar rats showed that 6-OHDA-induced PD symptoms were successfully reversed after the intranasal NE administration of naringenin + levodopa. A biochemical investigation revealed a significant increase in the levels of glutathione and superoxide dismutase, while the levels of malondialdehyde were significantly lowered in the test group treated with naringenin + levodopa [71].

Mustafa et al. (2012) developed the intranasal nanoemulsion of ropinirole, a dopamine agonist approved for use to treat symptoms of early and advanced PD. The isotropic area identified by a pseudoternary-phase diagram was used for formulation development. The optimized formulation contained 2 mg of ropinirole, along with Sefsol 218 (10% *v*/*v*), Tween 80 (18% *v*/*v*), Transcutol (18% *v*/*v*), and water (54% *v*/*v*) as the oily matrix, surfactant, cosurfactant, and aqueous phase, respectively. The optimized formulation with a globule size of 58.61 ± 5.18 nm depicted a cumulative drug release of 72.23 ± 9.56%, a viscosity of 31.42 ± 6.97 mpas, and infinite dispersion capability. The ex vivo study evidenced significantly high (*p* < 0.005) drug translocation to the Wistar rat brain in comparison to the drug itself. The authors concluded ropinirole-loaded NE as a promising perspective for the management of PD when administered intranasally [72].

Resveratrol is known for its clinical efficacy for reducing the production of amyloid peptides, in addition to the reduction of cognitive defects and its cytoprotective actions [73]. Resveratrol-loaded vitamin E NE was developed for brain-delivered modality in PD. The research group formulated a kinetically stable nanoemulsion (o/w) using vitamin E and propylene glycol mono-caprylic ester (Sefsol^®^) in a 1:1 ratio as the oil phase and Smix (Tween 80 as the surfactant and Transcutol P as the cosurfactant). Resveratrol NEs showed significantly high ex vivo mucosal flux across the porcine nasal mucosa in Franz diffusion cell assembly. Brain-targeting studies in Wistar rats demonstrated a higher drug concentration in the brain after the intranasal administration of resveratrol NEs. Furthermore, histopathological studies affirmed diminutive degenerative changes in the brain during the intranasal administration of resveratrol NE [70].

Despite the promising benefits of conventional NEs, lipidic NEs are preferred, as these require considerably lower amounts of surfactants and cosurfactants for their formulation. Lipid NEs are a relatively new drug delivery system prepared from soybean oil, triglyceride, and egg yolk lecithin [74]. An example is a hyaluronic acid-based mucoadhesive lipidic NE co-encapsulating two polyphenols for the nasal treatment of neurodegenerative diseases. Nasr (2016) co-encapsulated two polyphenols, curcumin and resveratrol (1:1 weight ratio), in mucoadhesive NEs made of hyaluronic acid for nose-to-brain targeting (Table 1). The optimized NE was subjected to an antioxidant potential assessment, in vitro and ex vivo release of both the polyphenols, in vivo quantification of the two drugs in rat brains, and safety on nasal mucosa. The optimized hyaluronic acid-based NE with a globule size of 115.2 ± 0.15 nm and a zeta potential of −23.9 ± 1.7 mV displayed higher mucoadhesive strength compared to its non-mucoadhesive counterpart, preserved the antioxidant ability of the two polyphenols, and conferred protection from degradation. In vivo studies evidenced about seven- and nine-fold increases in the AUC_(0–7 h)_ in the brain for resveratrol and curcumin, respectively, with respect to the drug given in the solution. Thus, hyaluronic acid-based lipidic NE was a successful carrier in enhancing the solubility, stability, and brain targetability of polyphenols.

Selegiline, an antioxidant and neuroprotective agent, is used for the oral therapeutics of PD, AD, depression, narcolepsy, and cocaine addiction. Of lately, it has been proposed as monotherapy for early stage PD [75] to hold back the treatment with levodopa to avoid its side effects for a substantial period. Other problems with the oral therapy of selegiline are poor drug bioavailability (<10%) due to its poor solubility in water and high presystemic metabolism. Owing to its poor access to the brain via oral administration, Kumar et al. investigated the intranasal NE of selegiline for direct nose-to-brain delivery [50]. The NE was formulated using Tween 80, Sefsol 218, and grape seed oil (1:1 by vol.). Grape seed oil has a synergistic antioxidant activity with selegiline, owing to omega-3 fatty acids that modulate the neuronal functions. Ex vivo permeation studies across the porcine nasal mucosa showed a higher flux than drug suspension. Selegiline being a substrate of P-glycoprotein could not diffuse across the nasal mucosa efficiently due to the presence of a P-glycoprotein efflux pump. The presence of P-glycoprotein in the nasal cavity may raise several challenges for drug absorption. It has been observed that P-glycoproteins are overexpressed in the nasal cavity, which comes from the respiratory mucosa in the cases of chronic rhinosinusitis. Hence, drug transport through nasal drug delivery may be affected during chronic rhinosinusitis [19]. However, in the presence of Tween 80 in the NE, a P-glycoprotein inhibitor, the efflux pump activity was reduced, thus allowing selegiline to diffuse across the porcine nasal mucosa. The efficacy of the NE formulation was affirmed by behavioral studies in a haloperidol-induced PD model in Wistar rats (Table 1).

NEs are also known to extend drug delivery from the nose to the brain via the olfactory region when mucoadhesive elements are added to the formulation. One such example is the report by Mustafa et al. (2015) wherein the brain-targeting potential of chitosan-coated oil in water NEs delivered intranasally in a haloperidol-induced PD rat model was investigated. The chitosan-coated NE containing a drug developed via the aqueous titration method followed by a high-pressure homogenization depicted a substantially high mucoadhesive potential in comparison to the conventional and homogenized formulations when monitored by gamma scintigraphy. Furthermore, the confocal study showed the deep localization of formulations in the brain confirming the BBB permeation potential of chitosan-coated NE. The pharmacokinetic data of the intranasal mucoadhesive NE in a Wistar rat brain and plasma indicated a significantly high (*p* < 0.005) AUC_0–24_ and amplified C_max_ (peak of maximum concentration) over the intravenous treatment group. Thus, the investigation demonstrated the potential of the intranasal delivery of mucoadhesive nanocarriers in the efficient management of PD [76].

### 4.3. NE for Migraines

Migraines, a headache disorder, are characterized by moderate-to-severe pain attacks that result in several autonomic dysfunctions like nausea, photophobia, vomiting, exertion, gastric stasis, small bowel, etc. [77]. Oral drug delivery is not suitable for migraine therapeutics primarily due to nausea and vomiting, which result in the poor gastrointestinal absorption of drugs [78]. Parenteral and nasal drug deliveries are thus best-suited for the treatment of migraines. A novel drug delivery system of Imitrex nasal spray (sumatriptan nasal spray) is indicated for the acute treatment of migraine attacks with or without auras in adults. It is not intended for the prophylactic therapy of migraines or for use in the management of hemiplegic or basilar migraines [79]. The delivery shows a rapid onset of action of the drug by deposition in the olfactory region and then travels from the nose to the brain.

Rizatriptan is a 5HT 1B/1D receptor agonist with a half-life of two to three h and an oral bioavailability of 40%. It is commercially available as tablets and orally disintegrating tablets. Though orally disintegrating tablets are suitable for administration during a migraine attack, it would not improve the poor bioavailability of the drug. To increase the bioavailability and brain tissue deposition of rizatriptan, its intranasal delivery systems were investigated for antimigraine therapy. Bhanushali et al. developed both intranasal NE and gel formulations of rizatriptan benzoate for controlled drug release and the direct targeting of the drug to the brain. The most important findings of the study, including improved brain targeting, are tabulated in Table 1. Likewise, zolmitriptan-loaded NE for intranasal administration was developed using various gel formulations. Studies showed a higher permeability through the nasal mucosa for zolmitriptan from NE formulation than that from the solution (Table 1). Both the reports show evidence for brain targeting, but none demonstrated effective antimigraine therapeutics.

### 4.4. NE for Epilepsy

Introduced in the early twentieth century, phenobarbital is widely used due to its reduced cost and high therapeutic efficacy [80]. Other antiepileptic drugs, such as phenytoin, ethosuximide, sodium valproate, carbamazepine, and some benzodiazepines, have been utilized as antiepileptic agents since 1970 [81]. New antiepileptic drugs introduced over the last decade include felbamate, topiramate, vigabatrin, gabapentin, levetiracetam, lamotrigine, tiagabine, oxcarbazepine, and zonisamide. These medications offer advantages over older drugs due to superior pharmacokinetic and pharmacodynamic effects [82]. Antiepileptic drugs are generally administered orally in the form of tablets, capsules, solution, suspension, and delayed-release tablets or capsules. Parenteral formulations are used as an alternative to the oral route in conditions requiring a rapid onset of action. Rectal, buccal, and sublingual routes are utilized that have their own sets of limitations. Approximately one-third of epileptic patients are resistant to medical therapy with commercial antiepileptic dosage forms [83]. The drug resistance could be due to the drug not reaching the target area or due to the low drug concentration in the brain. Extensive hepatic metabolism, drug–drug interactions, high plasma protein binding, and the presence of multidrug resistance efflux transporters in the GIT (gastrointestinal tract) and BBB could be the major factors that restrict the transport of antiepileptic drugs effectively to the CNS and for the low therapeutic concentration [84,85].

Direct delivery of drugs into the brain by intracerebral or intracerebroventricular routes are considered important approaches for the management of epilepsy, especially in drug-resistant patients [86]. Several studies have used nanoformulations for the targeted delivery of antiepileptic drugs to the receptor site via intravenous, oral, intranasal, and transdermal routes [87,88]. Only a few reports can also be found on the NE-based direct nose-to-brain delivery of antiepileptic drugs.

Phenytoin NE was developed with the composition of oil: surfactant: cosurfactant Labrafil M 2125 CS: cremophor RH 40: PEG 400 (polyethylene glycol 400) in the ratio of 2:1:1 for nose-to-brain delivery of the drug. The developed formulation was evaluated for the pharmacotechnical characteristics, sterility validation, and in vitro nasal toxicity. The NE exhibited a clear and stable formulation with a globule size < 20 nm and neutral zeta potential. The in vitro release study showed 100% release of the drug from nanoemulsion over a period of 48 h. The optimized formulation was devoid of nasal toxicity, sterilizable by aseptic filtration across 0.22 μm, and stable [89]. Though claimed suitable for intranasal delivery, preclinical studies are required to evidence an efficient nose-to-brain transport.

Intranasal amiloride-loaded mucoadhesive NEs for nose-to-brain delivery were formulated by Jain et al. The formulation ingredients included oils (Labrafac, Triacetin, oleic acid, Labrafil, IPM (Isopropyl myristate), and olive oil); surfactants (Labrasol, Tween 80, Tween 20, and Cremophor EL); and cosurfactants (Transcutol P and Plurol Oleique). The globule size ranged between 9.41 ± 1.23 and 10.71 ± 1.09 nm. The mucoadhesive NE was devoid of toxicity on sheep nasal mucosa and was adjudged safely for the intranasal administration of an antiepileptic drug [13,90]. The study needs to be extended for the pharmacokinetic and pharmacodynamic evaluations of the developed formulation.

### 4.5. NE for Psychosis

One of the first reports on the use of NEs for intranasal administration in the scientific literature was made by our research group, wherein the NEs were utilized to deliver the antipsychotic drug risperidone to the brain. The drug is commercially available as oral dosage forms (tablets and oral solutions) that have poor bioavailability owing to the first-pass hepatic metabolism. Risperidone NEs were prepared using Capmul MCM as the oily phase and Tween 80 as the surfactant. Additionally, mucoadhesive NE was prepared by adding chitosan to the NEs [91]. In vivo studies in Swiss albino rats with technetium (99mTc)-labeled NEs evidenced rapid and higher drug transport into the brain after the intranasal administration of the mucoadhesive NEs in comparison to the plain NE administered intranasally, intravenously, and as a solution [92]. Analogous results were obtained for NEs loaded with olanzapine. The promising reports of intranasal mucoadhesive NEs were correlated to the increased nasal retention time of the formulation due to the presence of mucoadhesive chitosan [93,94].

Ziprasidone hydrochloride is commercially available as oral capsules (Geodon^®^ and Zeldox^®^) that exhibit low bioavailability due to the extensive first-pass metabolism of drug. Bahadur and Pathak (2012) reported the formulation of buffered mucoadhesive NEs loaded with ziprasidone hydrochloride by the aqueous titration method using Capmul MCM, Labrasol, and transcutol as the oil, surfactant, and cosurfactant, respectively. A phosphate buffer of pH 8.0 was used as the aqueous phase to obtain clear NEs. The diffusion coefficient of the mucoadhesive NE was 1.79 times more than that of the NE itself. The pharmacodynamic studies showed remarkable positive results in the paw test (*p* < 0.05) and locomotor activity (*p* < 0.05). The optimized mucoadhesive NE was devoid of nasal ciliotoxicity in sheep nasal mucosa, concluding the development of the buffered mucoadhesive NE of ziprasidone hydrochloride, which can be safely administered by intranasal route [57].

Quetiapine fumarate (QTP) is an atypical antipsychotic drug administered orally as tablets. The oral therapy of quetiapine shows low bioavailability (5–15%) due to poor water solubility and an extensive first-pass effect [58]. Hence, alternative formulations are desirable. Oil-in-water NEs were investigated to target the drug directly into the brain following intranasal administration. Tween 80 was used as the surfactant, and cosurfactants of varying hydrophilic and lipophilic balance (HLB) values—namely, Transcutol P (HLB 4.2), Emalex (HLB 9.2), and PEG (HLB 12)—were investigated to study the effects of different HLB values on the phase behavior of the NE. The surfactant and cosurfactant were blended in different weight proportions from 1:3 to 3:1. Pseudoternary-phase diagrams of the NE system were composed of Capmul MCM as the oily phase, Smix, and water. A more than two-fold increase in drug release was recorded in QTP NE containing a cosolvent than that of the pure drug. The in vivo study for the tissue distribution in Wistar rats post the intranasal administration of quetiapine fumarate-loaded NE had a shorter *t*_max_ (time to reach C_max_) 1 h for the brain as compared with blood (2 h) that was possibly due to the direct nose-to-brain transport through the olfactory region of the nose. Furthermore, a higher C_max_ value for intranasal quetiapine NE (0.48 ± 0.16 μg/mL) than pure drug intranasal administration (0.12 ± 0.83 μg/mL) illustrated the superiority of NE for nose-to-brain delivery. An around two-fold increase in the drug-targeting efficiency (DTE%) of NE as compared to the pure drug (from 140.83% ± 2.61% to 267.98% ± 3.06%) and more than two-fold increase in direct nose-to-brain transport (DTP%) (from 29.44% to 63.63%) quantified the transport efficiency of the NE formulation.

### 4.6. NE for CNS Infection

CNS infections entail encephalitis, meningitis, and brain abscesses and tend to cause higher morbidity and mortality on an average than infections of other organ systems. Other infectious diseases of the CNS, such as Prion disease and cavernous sinus thrombosis, are rare yet devastating infections [95]. The Human Immunodeficiency Virus (HIV) is capable of remaining dormant confined in the brain for years, together protected from antiretroviral drugs by the BBB [96]. After the initial infection, the CNS serves as an “anatomic reservoir” for HIV, from which the infection gets reactivated when triggered. The brain infection from HIV can manifest itself as neuro-AIDS (acquired immunodeficiency syndrome), a form of HIV-associated dementia and neurocognitive impairment. Thus, the improved drug delivery of anti-HIV drugs to the CNS will potentially reduce the possibility of relapse of the persisting infection. A protease inhibitor, saquinavir mesylate belongs to BCS (Biopharmaceutical Classification System) class II drug, with activity against HIV Type 1 has poor bioavailability (4%) owing to its poor aqueous solubility. Furthermore, the drug has poor permeability across the BBB and is a P-glycoprotein and cytochrome P450 3A4 isozyme substrate. All these reasons justify the development of intranasal o/w NE of saquinavir mesylate. The NEs were prepared by dissolving the drug (500 mg/mL) in a mixture of Capmul MCM (4–8%), Tween 80 (6–15.75%), and PEG 400 (2–5.25%) that was slowly added into water with stirring at 300 rpm. The optimized formulation showed 76.96% ± 1.99% (three-fold enhancement) in drug permeation from the NE as compared to plain drug suspension across sheep nasal mucosa in ex vivo studies. The results of biodistribution studies in Sprague Dawley rats showed a higher drug concentration in the brain (approximately 36 times) and plasma (about three-fold) after the intranasal administration of drug-loaded NE as compared to its intravenous administration. Thus, the intranasal administration allowed drug uptake into the CNS. The presence of the drug in the plasma is expected, since this route can also lead to systemic drug absorption, but the measured plasma concentrations were well below those found in the brain. Based on the AUC data determined over a 0–180-min period, the bioavailability of the drug via nasal NE was found to be 42.49% for the doses examined. This was correlated to a rapid absorption and longer residence time of the NE in the nasal cavity that provided the opportunity for intranasal delivery to the brain. The scintigrams confirmed major radioactivity accumulation in the brain following intranasal administration [97].

Another protease inhibitor, indinavir, used in the treatment of HIV infection has a limited efficacy in eradicating the HIV virus from the brain due to efflux by P-glycoprotein (P-gp) expressed at the BBB. Thus, an o/w lipid nanoemulsion (LNE) of indinavir was researched to improve its brain delivery using Tween 80 as a cosurfactant. LNEs were characterized for pharmacotechnical parameters, and selected formulations were subjected to brain uptake, pharmacokinetic, and tissue distribution studies. The brain uptake of indinavir was higher from the NE containing 1% Tween 80 compared to a formulation containing 0.3% cholesterol. Likewise, pharmacokinetic studies revealed a significantly higher (*p* < 0.05) brain level of indinavir from the formulation made of Tween 80 than produced by a drug solution (2.44-fold) or cholesterol-containing NE (1.6-fold). The authors explained that the increased brain-specific accumulation of indinavir was probably due to enhanced low-density lipoprotein-mediated endocytosis and P-gp inhibition by Tween 80 at the BBB. Thus, LNEs can be seen as simple and effective means of the brain-specific delivery of indinavir to the brain [98]. NEs loaded with chloramphenicol aimed at the treatment of meningitis were formulated using palm kernel oil esters. While the researchers presented the pharmacotechnical evaluation, they did not evidence a possible intranasal use of the developed formulations [99].

### 4.7. NE for Senile Dementia and Cerebrovascular Spasms

Most types of dementia cannot be cured, but there are ways to manage the symptoms. The drugs used to improve dementia symptoms are cholinesterase inhibitors (donepezil, rivastigmine, and galantamine) and memantine [100]. The donepezil and rivastigmine intranasal NEs for direct nose-to-brain delivery that can be used for depression therapeutics were described earlier. The usefulness of nimodipine in patients with AD and vascular dementia is still questionable but is frequently prescribed for cognitive impairment and dementia [101]. Pathak et al. developed nimodipine-loaded NEs for the potential treatment of senile dementia and cerebrovascular spasms [102]. Nimodipine has poor oral bioavailability (5–10%) due to a low aqueous solubility and extensive first-pass metabolism, and consequently, the drug achieves a low brain concentration upon oral administration. Furthermore, nimodipine is a P-glycoprotein substrate that can be another reason for low brain drug levels. For all these reasons, in situ gelling mucoadhesive NEs (4% Capmul MCM as the oil, 30% Labrasol as the surfactant, and Transcutol P as the cosurfactant; 2:1) were developed for the intranasal delivery of nimodipine. Polymers, Carbopol 934 P, chitosan, sodium alginate, and sodium CMC were tested as mucoadhesive agents, and Pluronic F 127 and Pluronic F 68 were screened as gelling agents. Sufficient gelling was obtained with 20% (*w/w*) Pluronic F 127 combined with 8% (*w/w*) Pluronic F 68 at physiological temperatures. An ex vivo drug permeation study across freshly excised goat nasal mucosa showed higher drug permeation (seven-fold) from the NE compared to drug suspension without toxicity to the nasal membrane. Pharmacokinetic studies in rats demonstrated higher brain concentrations of nimodipine (about 1.5 times) from in situ gelling systems as compared to NE and about 10-fold higher than the nimodipine suspension, all administered intranasally [102].

### 4.8. NE for Depression

Depression therapy includes various classes of drugs, including older-type (tricyclic antidepressants and monoamine oxidase inhibitors) and newer-type antidepressants. The new antidepressant classes are selective serotonin reuptake inhibitors (SSRIs), serotonin and norepinephrine reuptake inhibitors (SNRIs), norepinephrine, dopamine reuptake inhibitors [103], and esketamine. Esketamine was approved by the FDA in March 2019 for adults with treatment-resistant depression and is available as a nasal spray [104].

Upon oral administration, paroxetine shows a low bioavailability (less than 50%) due to the first-pass effect. In order to facilitate the passage of paroxetine across the BBB, o/w NEs for nose-to-brain targeting were developed by the spontaneous emulsification technique using Capmul MCM, Solutol HS 15, and propylene glycol as the oil phase, surfactant, and cosurfactant, respectively. A pharmacotechnical assessment identified the optimized formulation as spherical globules with a mean diameter of 58.47 ± 3.02 nm, polydispersity index (PDI) of 0.339 ± 0.007, zeta potential value of −33 mV, transmittance of 100.60% ± 0.577%, refractive index 1.412 ± 0.003, and viscosity of 40.85 ± 6.40 cP. Ex vivo studies across porcine nasal mucosa revealed three-fold enhancement in the drug permeation in comparison to the paroxetine suspension that was reflected in behavioral activities. Histopathological studies revealed decreased damage and degeneration of the vesicular nuclei of the brain tissues of Wistar rats affirming the targeted delivery of the drug to the brain [105].

In another research, the formulation of sertraline hydrochloride NE for intranasal delivery of the drug was aimed to achieve a rapid onset of action, to minimize the first-pass effect, and to enhance its bioavailability. The sertraline hydrochloride NE system with Capmul MCM as the oil phase, Labrasol as the surfactant, and Transcutol P as the cosurfactant was assessed for the solubilization capacity and phase behavior. It was noted that the NE enhanced the solubility of the drug to 94.28 mg/mL from approximately 0.5 mg/mL, and the in vitro permeation across the goat mucosa was 62.8% ± 0.56%. The results suggested the potential benefits of intranasal delivery over the available oral delivery (tablet) for the treatment of depression [106]. However, in vivo studies are required to prove the results of the ex vivo studies.

### 4.9. NE for Brain Tumors

Brain tumors are challenging to treat due to the BBB obstacle and the fact that tumors are frequently diagnosed late [107]. Owing to the fact that brain tumors exhibit many distinctive features relative to tumors growing in peripheral body tissues, targets based on continuously changing vascular characteristics and the microenvironment of tumors can be utilized to facilitate efficient brain tumor-targeted drug delivery [108]. Of over 120 types of brain tumors, the two most common types are meningioma and glioma [109]. Glioblastoma is the most dangerous form of brain tumor, characterized by rapid growth and invasion of the surrounding tissue. Currently, the standard clinical treatment for glioblastoma is surgery, followed by radiotherapy and concurrent chemotherapy with temozolomide [110]. Although extensive research efforts have been dedicated in the past to develop an effective therapeutic strategy for the treatment of glioblastoma, major improvements in terms of the overall survival of patients is yet to be achieved. Hence, novel therapeutic strategies are urgently required. Many formulations based on nanosized and nanostructured drug delivery systems have been investigated for enhancing the nose-to-brain transport. The drugs researched for nose-to-brain delivery via NEs for treating brain tumors are listed in Table 2.

The augmentation of therapeutic activity with a combination of paclitaxel and C(6)-ceramide, when administered as o/w NEs, was examined in human glioblastoma cells. The paclitaxel and ceramide coloaded NE formulated with pine nut oil had an oil droplet size of approximately 200 nm in diameter. When administered to U-118 cells, a significant increase in cytotoxicity was observed with the amalgamation of paclitaxel and ceramide NE as compared to the administration of solo agents. The enhancement in cytotoxicity was correlated to an increase in apoptotic activity in cells due to the amalgamation of paclitaxel and ceramide coloaded in NEs. The results of the study present an efficient option to design o/w NEs for the combination therapy of brain tumor cells, especially in aggressive tumor models such as glioblastoma [114].

In another study, 5-fluorouracil NEs for targeting brain tumors were formulated, optimized, and characterized. Based on the results of the characterization, the optimized NE was selected for conjugation with the epidermal growth factor by two different methods—namely, the physical mixing and solvent evaporation technique. The physical mixing technique was optimized as the best method for conjugating the formulation. The targeting efficiency of the drug was studied by molecular docking for the conjugated drug formulation, and it was deduced that the binding energy level of the drug-conjugated formulation with the epidermal growth factor was high when compared with the plain drug [115].

Recently, theranostic drugs that are simultaneously used as a biomarker and remedy for neoplastic diseases have gained prominence. Nanoparticles, and particularly NEs, have the caliber to accentuate therapeutic drug concentrations at the target site, thereby enhancing the drug potency, minimizing the drug toxicity and side effects, and achieving a sustained drug concentration over prolonged periods of time [116]. Magnetic materials can also be incorporated into NEs to produce magnetic nanoemulsions (MNEs) to perform precise biological functions. The o/w NE can be developed as MNEs with superior thermodynamic stability [117]. The oily phase in NEs offers an optimal chemical arena to host phthalocyanine compounds with lipophilic characteristics. Paula et al. reported the development of MNEs loaded with chloroaluminumphthalocyanine and citrate-decorated maghemite nanoparticles. An in vitro test was performed to assess the cell viability using the BM-MSC, U87MG, and T98G cell lines. The cells were incubated with the two prepared formulations before and after performing a hyperthermia treatment (40-Oe magnetic field amplitude and 1-MHz frequency) and photodynamic therapy (700-mJ/cm^2^ energy density and 670-nm wavelength). For all cell lines subjected to hyperthermia treatment, the decrease of the cell viability averaged to 15%, regardless of the magnetic nanoparticle content in the MNE. On the other hand, on using both the MNE formulation and photodynamic therapy, an average decrease of 52% was achieved. Furthermore, a 70% decrease was achieved on integrating the hyperthermia and photodynamic therapy treatments. Confocal laser scanning microscopy clearly traced out the cytoplasm localization and the active site of the MNEs. Thus, the collaborative treatment of photodynamic therapy and hyperthermia treatment represents a promising paradigm for brain carcinoma treatment with MNEs [118].

In an interesting study, commercially available Total Parenteral Nutrition NEs—namely, Intralipid^®^ (Fresenius Kabi, Bad Homburg, Germany) and Clinoleic^®^ (Baxter Healthcare, Deerfield, IL, USA), were used as the nanovehicles and solubilizers of paclitaxel. The formulation efficacy was evaluated against glioma and normal glial cells. The globule size of the NEs was highly dependent on the NE type. Hence, the sizes varied in the range of 254–264 nm for Clinoleic^®^ and 283–295 nm for Intralipid^®^, depending on the concentration of the drug. The drug entrapment varied in the 70–80% and 44–57% ranges for the Clinoleic^®^ and Intralipid^®^ formulations, respectively. In the cell line studies, paclitaxel-loaded Clinoleic^®^ NE decreased U87-MG glioma cells’ viability to 6.4%, compared to only 21.29% using Intralipid^®^ NE. Both NEs were less cytotoxic to normal glial cells (SVG-P12) in comparison to the glioma cell line, indicating the specificity of the NEs against malignant cancer cells. While the authors proved the efficacy of drug-loaded NEs against glioblastoma cell lines, the nose-to-brain transport of the formulations needs to be addressed [119].

Interestingly, drug moiety-loaded carriers devoid of targeting ligands have been demonstrated to exert chemotherapeutic effects in orthotopic brain glioma models [120,121], that pave the way for the clinical therapy of brain carcinomas. Taxotere^®^ (Sanofi-Aventis, Bridgewater Township, NJ, USA), the commercial formulation of docetaxel, is toxic and is nonselectively distributed; thus, novel formulations with less deleterious and enhanced tumor targeting are desirable. A docetaxel-loaded NE with 72.3-nm globule size, −6.38 mV zeta potential, 2.87% drug loading capacity, and 93.1% encapsulation efficiency was formulated. Although the docetaxel-loaded NE presented similar antiproliferative effects on U87 cells and bEnd.3 cells, its in vivo toxicity was significantly lower than that of Taxotere^®^. Fluorescent imaging in rats suggested NE-loaded fluorescent probes could preferentially distribute in the brain and localize at the glioma site. The pharmacological experiments also confirmed that the docetaxel-loaded NE could target glioma sites by prolonging the median survival time of mice bearing gliomas [122].

### 4.10. NE for Neuroprotection

Neuroprotection is the therapy that can confer protection to dopaminergic neurons of the midbrain. Though several neuroprotective agents have been screened in clinical trials, none of them have passed as of now [123]. The neuroprotective effects of an immunosuppressant drug cyclosporine-A can be attained but only with very high oral doses that result in side effects, such as hepatotoxicity, immunosuppression, and nephrotoxicity. Hence, cyclosporine-A administered via the oral route as a neurotherapeutic is not a choice. The study by Yadav et al. demonstrated that the intranasal administration of NEs loaded with cyclosporine-A is an efficient approach to target the brain in comparison to intravenous administration that does not efficiently transport cyclosporine-A across the BBB. The o/w NEs of cyclosporine-A were formulated by the ultrasonication method, wherein a prewarmed oil phase consisting of flaxseed oil and cyclosporine-A dissolved in ethanol was gradually added to the prewarmed aqueous phase comprising of lipoid E80, Tween 80, and stearylamine. The optimized formulation constituted of 20% flaxseed oil, 2.4% *w*/*v* lipoidE80, 0.2% *w*/*v* Tween 80, and 0.2% *w*/*v* stearylamine. The uniform milky white NE with a total drug concentration of 25 mg/mL had 88% ± 13% encapsulation efficiency. The researchers compared the uptake of cyclosporine-A in the brain regions and peripheral organs following the intranasal and intravenous administration of cyclosporine-A NE and its solution. The intranasal administration of NE resulted in a higher accumulation compared to any other treatment and route of administration. Following the intranasal administration of cyclosporine-A NE, the maximum concentrations obtained in the olfactory bulb, midbrain, and hindbrain were similar, in the range of 200−300 ng/g of the brain tissue. However, the kinetics were different in the three brain parts. The peak concentration was obtained in the olfactory bulbs after one h versus four h in the midbrain, suggesting a rostral-to-caudal gradient over time. The trend in the hindbrain was unclear. The brain/blood drug ratios of 4.49, 0.01, 0.33, and 0.03 for the NE (IN, intranasal), NE (IV, intravenous), solution (IN), and the solution (IV), respectively, indicated direct nose-to-brain transport of the drug through NE, bypassing the BBB. Furthermore, the intranasal NE reduced the nontarget organ exposure [55].

In another study, Yadav et al. assessed the therapeutic use of intranasal cationic NE-encapsulated siRNA, an antitumor necrosis factor-alpha (TNF-α), for potential anti-inflammatory therapy for the brain. Flaxseed oil containing cationic NE formulations encapsulating a TNFα-silencing siRNA duplex were formulated using the cationic lipid DOTAP (*N*-(1-[2, 3-dioleoyloxy] propyl)-*N*,*N*,*N*-trimethylammonium salt). After complexation of the siRNA duplex with DOTAP, flaxseed oil was gradually added to the mixture. An aqueous phase containing Lipoid^®^ E80 and Tween^®^ 80 was then subsequently added dropwise to the oil phase. The resultant mixture was homogenized to get NE. Since the substantia nigra in the brain is one of the major targets for neuroinflammation diseases, the researchers selected the middle-brain region at the bregma coordinates of 4.70 to 7.80 mm to study the uptake of therapeutic siRNA. The siRNA NE showed a higher (1.38%) injected dose in the middle-brain region at the six-hour time point compared to the 0.2% injected dose at the six-hour time point delivered intranasally in a saline solution in Wistar rats [124].

### 4.11. NE for Multiple Sclerosis and Amyotrophic Lateral Sclerosis

Multiple sclerosis is a complex neurological disorder and manifests itself as various clinical symptoms, such as visual problems, changes in sensation, muscle weakness, and difficulty in speech and coordination [125]. In the past, several investigations have been carried out to introduce novel therapeutic approaches for multiple sclerosis patients. Among the current multiple sclerosis drugs, interferon-β was marketed first. Subsequently, glatiramer acetate was introduced, followed by natalizumab, fingolimod (first oral drug), and teriflunomide. These are the latest drugs marketed for multiple sclerosis with a sufficient evidence of efficacy [126]. However, the clinical use of these drugs is associated with a plethora of side effects. Some FDA-approved NE formulations such as Camptosar and Fluosol have been evaluated in multiple sclerosis patients [127]. It should be noted that intranasal NEs have not been investigated for multiple sclerosis to date, and the area offers immense research options.

Riluzole, a BCS class II drug, is approved to treat amyotrophic lateral sclerosis. Even though it has an absolute bioavailability of 60%, riluzole is a substrate of P-glycoprotein and cannot cross the BBB to reach the brain. To achieve direct brain delivery, riluzole-loaded NEs were formulated using the phase titration method. The best formulation consisted of 3% *w*/*w* Sefsol 218, 28.3% *w*/*w* Tween 80:Carbitol (1:1), and 68.7% *w*/*w* water. With an average globule size of about 24 nm, the NE was free from nasal ciliotoxicity and was stable for three months. The brain uptake of the drug following the intranasal route of riluzole NEs was significantly higher (*p* < 4.10 × 10^−6^) compared to oral administration of the NE. Thus, intranasal riluzole NE could be a promising approach for the treatment of amyotrophic lateral sclerosis to minimize the dose of riluzole [128].

### 4.12. NE for Cerebral Ischemia

Oxidative stress is the foremost factor that intensifies the damage of cerebral ischemia [129]. Thymoquinone has antioxidant properties and, perhaps, could be a remedy for cerebral ischemia [130], but due to low solubility (<1.0 mg/mL at room temperature) and poor absorption, it exhibits low serum and tissue levels after oral administration. Thymoquinone mucoadhesive NEs with a 100-nm mean size were prepared by the ionic gelation method using oleic acid as the oily phase, Tween-20/Carbitol as the surfactant/cosurfactant, and chitosan as the mucoadhesive agent for improving the bioavailability. An increase in the drug permeability (1.7-fold) across goat nasal mucosa in vitro confirmed the penetration-enhancing properties of chitosan used in the formulation. An in vivo study in Wistar rats exhibited an enhanced drug bioavailability in the brain after intranasal administration compared to intravenous administration. A pharmacokinetic study revealed a significantly higher AUC_0–t_ after intranasal administration of the developed NE compared to pure thymoquinone in all three tissues (brain, lungs, and plasma). The brain/plasma ratio was higher (10.66) via the intranasal route with respect to intravenous administration (0.22) for the mucoadhesive NE. The brain-targeting efficiency of 628.5786 ± 44.79% and the brain drug-targeting potential of 89.97% ± 2.94% confirmed the brain-specific delivery of thymoquinone [131].

## 5. Current Challenges and Future Prospects

CNS disorders are most challenging to treat due to the reasons emphasized in the above text. Additionally, many diseases are refractory to small molecule treatments, and still, for most of the diseases, a cure is a distant dream. There are certain brain disorders that still need the attention of researchers. For instance, Huntington’s disease is a genetic neurodegenerative disorder that affects cognitive and motor abilities. Tetrabenazine is currently the only drug approved by the FDA for the symptomatic treatment of Huntington’s disease. The drug is given orally and has a relatively low bioavailability of 4.9% [132]. The intranasal formulation of the drug may improve its bioavailability. Likewise, there are many other therapeutic molecules that can be subjected to intensive research. While compiling this review, many research gaps were identified that have been mentioned at appropriate places in the text. Many research reports that aimed at intranasal administration did not prove the same results either by pharmacokinetic or pharmacodynamic studies. In such cases, in vitro BBB models may be used as surrogates to animal studies in-line with the 3Rs (Replacement, Reduction and Refinement) principle.

While intranasal nanoemulsions can be a promising approach for direct drug delivery to the brain, the efficacy of this route is subjected to many determinants. The foremost factors that determine the efficacy of delivery via this route include effective delivery to the olfactory region of the nares, extended retention time across the surface of the nasal mucosal, a reduction in drug metabolism in the nasal cavity, and enhancing the active penetration through the nasal epithelia [133].

Nasal mucociliary clearance is a major challenge that needs to be addressed. Mucoadhesive formulations of NEs can be the answer. Another concern of intranasal NEs is related to the irritation and damage of nasal mucosa on repeated administrations of NEs. Studies have shown that surfactants are good permeation enhancers that may cause irreversible damage of the nasal mucosa [134]. These damages can affect the nasal septum and may cause bleeding and irritation when administered repeatedly. The toxicity studies in the research reports are either short-term (2–4 h) or single-dose ex vivo studies on the nasal mucosa, while the treatment of CNS diseases like epilepsy, PD, Alzheimer’s disease, and many others may continue for a lifetime once diagnosed. Hence, future research should be more focused on long-term toxicity studies in animals.

The total drug dose delivered to the site of the drug action is the key endpoint for effective pharmaceutical action. In brain delivery, the total dose not only relies on the total amount of drug delivered via the olfactory region, but it also necessarily depends on the transfer and distribution of the drug within the brain. As the brain is a dynamic organ, the biodistribution of the drug within the brain and its relation to an effective dose could be an emerging research area [8]. The computational assessment of drug delivery to the brain via the olfactory region has been primarily focused on simulations of airflow during inhalation, focusing on the motion of gases, particles, and droplets and their deposition onto the olfactory mucosa. Other considerations are the drug release, the interaction of deposited entities with the mucus layer, and the transfer of drug molecules across the mucus layer, all of which have hardly been investigated. There is an urgent need for robust computational models to describe these aspects to obtain a better understanding of delivery to the brain and, also, to assist in the design, execution, and analysis of existing and future experimental studies [135,136,137].

## 6. Conclusions

NEs are a promising approach for the nose-to-brain delivery of drugs to reach the desired concentrations of therapeutics in the brain. Several in vivo studies have been reported to validate the effectiveness of NEs for nose-to-brain delivery for neuronal diseases at the preclinical level. However, extensive studies are still required for the safety and toxicity-related issues of NEs before clinical applications of the delivery systems. Indications where nose-to-brain products are likely to emerge first commercially include the neurodegeneration, pain, and glioblastoma.

## Figures and Tables

**Table 1:** Summarized examples of nanoemulsion-based approaches for brain targeting through intranasal drug delivery.

Drug	Therapy for	Characterization Parameters	Study Model (s)	Relevant Therapeutic Outcomes	Ref.
Donepezil	Alzheimer’s disease	GS = 127.13 ± 4.14 nm PDI = 0.182 ± 0.011	In vitro drug diffusion study. Ex vivo drug permeation study. Tolerability study through in vitro and in vivo models.	The permeation of donepezil was found to be significant through intranasal NE. The polymers can be used as an effective strategy to improve the bioadhesion and drug penetration through nasal mucosa, which enhances the bioavailability of donepezil.	[47]
Rivastigmine	Alzheimer’s disease	GS = 35.75 ± 0.21 nm PDI = 0.247 ± 0.04 ZP = −24.4 ± 0.67 mV	In vitro drug release study. Ex vivo diffusion study. In vivo pharmacokinetic and biodistribution study in rat. Nasal ciliotoxicity studies in goat nasal mucosa.	Rivastigmine-loaded NE showed significantly higher drug concentration in brain than the solution. The optimized formulation was devoid of nasal ciliotoxicity.	[48]
Resveratrol	Parkinson’s disease	GS = 176.3 ± 3.5 nm PDI = 0.17 ± 0.03 ZP = 18.5 ± 1.77 mV	In vitro drug release study. Ex vivo diffusion study. In vivo drug biodistribution study in Wistar rat’s brain.	Diffusion controlled release of resveratrol was for 6 h with flux of 2.86 mg/cm^2^ h through sheep nasal mucosa. The drug level in the brain from intranasal resveratrol mucoadhesive NE was higher than the resveratrol solution. Bioavailability was seven times higher through this approach.	[49]
Selegiline	Parkinson’s disease	GS = 61.43 ± 4.10 nm PDI = 0.203 ± 0.005 ZP = −34.00 ± 0.17 mV	In vitro drug release study. Ex vivo diffusion study. Behavioral activities of Parkinson’s disease in Wistar rats.	Selegiline NE showed 3.7-fold more penetration than the drug solution. Haloperidol-induced Parkinson’s disease in animals with selegiline intranasal NE showed significant improvement in behavioral activities in comparison to conventional drug delivery.	[50]
Letrozole	Epilepsy	GS = 95.59 ± 2.34nm PDI = 0.162 ± 0.012 ZP = −7.12 ± 0.12 mV	In vitro and ex vivo drug release study. A behavioral seizure; biochemical and histopathological studies were performed.	Intranasal administration of NE showed the prolonged drug release profile as compared to suspension. High concentration of drug was found in brain.	[51]
Amiloride	Antiepileptic	GS = 89.36 ± 11.18 nm PDI = 0.231 ± 0.018 ZP = −9.83 ± 0.12 mV	In vitro drug release study. Ex vivo diffusion study. In vivo pharmacodynamic and pharmacokinetic study in Wistar rats.	Bioavailability and brain-targeting efficiency with efficacy of developed amiloride NE was enhanced though nasal administration.	[52]
Zolmitriptan	Migraine	GS = 54.63 ± 3.24 nm ZP = −0.086 ± 0.014 mV PDI = 0.17 ± 0.01	In vitro mucoadhesion study. Ex vivo drug permeation studies. In vivo pharmacokinetic and biodistribution studies.	Zolmitriptan mucoadhesive NE showed higher permeability coefficients than the solution through the nasal mucosa. In vivo study of zolmitriptan mucoadhesive NE showed higher AUC_0–8_ and shorter T_max_ in the brain in comparison to intravenous and nasal solutions.	[53]
Rizatriptan	Migraine	GS = 20–120 nm	In vitro drug diffusion study. Nasal irritation study on sheep nasal mucosa. In vivo brain-targeting potential.	Ex vivo drug diffusion-defined controlled release with 86% in 4 h. Brain targeting through intranasal NE (AUC = 302.52 μg min/g) was more than intranasal gel (AUC = 115 μg min/g) and intravenous route (AUC = 109.63 μg min/g).	[54]
Cyclosporine-A	Neuroprotective	GS = 158.47 ± 3.02 nm ZP = −30 mV	In vitro drug diffusion study. In vivo brain uptake study.	The brain/blood ratios of cyclosporine-A by intranasal and intravenous was found to be 4.49 and 0.01, respectively. Cyclosporine-A NE can be used for direct nose-to-brain delivery, bypassing the BBB.	[55]
Kaempferol	Neuroprotective and anti-tumor	GS = 170.4 ± 4.1 nm PDI = 0.155 ± 0.015 ZP = −18.71 ± 1.72	Ex vivo diffusion study. In vivo drug biodistribution study in Wistar rats.	The drug concentration through intranasal NE was found to be 4 to 5-fold higher than the solution. Ex vivo permeation and in vivo biodistribution studies showed higher drug concentrations in the brain with chitosan NE through intranasal administration in compared to NE and the kaempferol solution.	[56]
Ziprasidone hydrochloride	Antipsychotic	GS = 145.24 ± 4.75 nm PDI = 0.186 ± 0.40 ZP = −30.2 ± 3.21 mV DC = 0.3418 ± 0.03 CM^2^/min	Ex vivo diffusion study. In vivo pharmacodynamic study in Wistar rats. Nasal ciliotoxicity studies in goat nasal mucosa.	Higher drug diffusion of ziprasidone NE than the solution was found. Pharmacodynamic study revealed the superiority of mucoadhesive NE than NE in the locomotor activity and paw test. Formulation was devoid of acute nasal ciliotoxicity.	[57]
Quetiapine	Antipsychotic	GS = 144 ± 0.5 nm	In vitro dissolution study. In vivo drug distribution study in Wistar rats.	Higher drug transport efficiency (DTE%) via intranasal NE.	[58]

Abbreviations: GS = globule size, PDI = polydispersity index, ZP = zeta potential, DC = diffusion coefficient, NE = nanoemulsion, BBB = blood–brain barrier, and AUC = area under the curve.

**Table 2:** The drugs researched for brain tumor therapeutics via intranasal nanoemulsions.

Drug	Method of Preparation	GS and ZP	In Vivo Model	Ref.
Ecto-50-nucleotidase (CD73)	Microfluidization	262.7 ± 12.8 nm +3.5 ± 3.0	C6 rat glioma	[111]
Kaempferol	High-pressure homogenization	180.53 ± 4.90 nm (coated) +26.09 ± 2.67 (coated) 145.07 ± 4.91 nm (uncoated) −18.10 ± 2.55 (uncoated)	N/A	[112]
Temozolomide	High-pressure homogenization	134 nm −13.11	N/A	[113]

## References

[1] Martins P.P., Smyth H.D., Cui Z. (2019). Strategies to facilitate or block nose-to-brain drug delivery. Int. J. Pharm..

[2] Pardridge W.M. (2005). The blood-brain barrier: Bottleneck in brain drug development. NeuroRX.

[3] Gadhave D., Choudhury H., Kokare C.R. (2019). Neutropenia and leukopenia protective intranasal olanzapine-loaded lipid-based nanocarriers engineered for brain delivery. Appl. Nanosci..

[4] Pardeshi C.V., Belgamwar V.S. (2013). Direct nose to brain drug deliveryviaintegrated nerve pathways bypassing the blood–brain barrier: An excellent platform for brain targeting. Expert Opin. Drug Deliv..

[5] Hada N., Netzer W.J., Belhassan F., Wennogle L.P., Gizurarson S. (2017). Nose-to-brain transport of imatinib mesylate: A pharmacokinetic evaluation. Eur. J. Pharm. Sci..

[6] Bonaccorso A., Gigliobianco M.R., Pellitteri R., Santonocito D., Carbone C., Di Martino P., Puglisi G., Musumeci T. (2020). Optimization of Curcumin Nanocrystals as Promising Strategy for Nose-to-Brain Delivery Application. Pharmaceutics.

[7] Fahmy U.A., Badr-Eldin S.M., Ahmed O.A.A., Aldawsari H.M., Tima S., Asfour H.Z., Al-Rabia M.W., Negm A.A., Sultan M.H., Madkhali O.A.A. (2020). Intranasal Niosomal In Situ Gel as a Promising Approach for Enhancing Flibanserin Bioavailability and Brain Delivery: In Vitro Optimization and Ex Vivo/In Vivo Evaluation. Pharmaceutics.

[8] Bourganis V., Kammona O., Alexopoulos A., Kiparissides C. (2018). Recent advances in carrier mediated nose-to-brain delivery of pharmaceutics. Eur. J. Pharm. Biopharm..

[9] Shingaki T., Hidalgo I.J., Furubayashi T., Sakane T., Katsumi H., Yamamoto A., Yamashita S. (2011). Nasal delivery of P-gp substrates to the brain through the nose-brain pathway. Drug Metab. Pharmacokinet..

[10] Choudhury H., Gorain B., Chatterjee B., Mandal U.K., Sengupta P., Tekade R.K. (2017). Pharmacokinetic and Pharmacodynamic Features of Nanoemulsion Following Oral, Intravenous, Topical and Nasal Route. Curr. Pharm. Des..

[11] Illum L. (2000). Transport of drugs from the nasal cavity to the central nervous system. Eur. J. Pharm. Sci..

[12] Illum L. (2004). Is nose-to-brain transport of drugs in man a reality?. J. Pharm. Pharmacol..

[13] Bonferoni M.C., Rossi S., Sandri G., Ferrari F., Gavini E., Rassu G., Giunchedi P. (2019). Nanoemulsions for “Nose-to-Brain” Drug Delivery. Pharmaceutics.

[14] Rassu G., Soddu E., Cossu M., Brundu A., Cerri G., Marchetti N., Ferraro L., Regan R.F., Giunchedi P., Gavini E. (2015). Solid microparticles based on chitosan or methyl-β-cyclodextrin: A first formulative approach to increase the nose-to-brain transport of deferoxamine mesylate. J. Control. Release.

[15] Mistry A., Stolnik S., Illum L. (2015). Nose-to-Brain Delivery: Investigation of the Transport of Nanoparticles with Different Surface Characteristics and Sizes in Excised Porcine Olfactory Epithelium. Mol. Pharm..

[16] Quintana D.S., Guastella A.J., Westlye L.T., Andreassen O. (2015). The promise and pitfalls of intranasally administering psychopharmacological agents for the treatment of psychiatric disorders. Mol. Psychiatry.

[17] Wu H., Hu K., Jiang X. (2008). From nose to brain: Understanding transport capacity and transport rate of drugs. Expert Opin. Drug Deliv..

[18] Agrawal M., Saraf S., Saraf S., Antimisiaris S.G., Chougule M.B., Shoyele S.A., Alexander A. (2018). Nose-to-brain drug delivery: An update on clinical challenges and progress towards approval of anti-Alzheimer drugs. J. Control. Release.

[19] Bors L.A., Bajza Á., Mándoki M., Tasi B.J., Cserey G., Imre T., Szabó P., Erdő F. (2020). Modulation of nose-to-brain delivery of a P-glycoprotein (MDR1) substrate model drug (quinidine) in rats. Brain Res. Bull..

[20] Mittal D., Ali A., Md S., Baboota S., Sahni J.K., Ali J. (2013). Insights into direct nose to brain delivery: Current status and future perspective. Drug Deliv..

[21] Casettari L., Illum L. (2014). Chitosan in nasal delivery systems for therapeutic drugs. J. Control. Release.

[22] Gupta S., Kesarla R., Omri A. (2019). Approaches for CNS delivery of drugs—Nose to brain targeting of antiretroviral agents as a potential attempt for complete elimination of major reservoir site of HIV to aid AIDS treatment. Expert Opin. Drug Deliv..

[23] Ruigrok M.J., De Lange E.C. (2015). Emerging Insights for Translational Pharmacokinetic and Pharmacokinetic-Pharmacodynamic Studies: Towards Prediction of Nose-to-Brain Transport in Humans. AAPS J..

[24] Crowe T.P., Greenlee M.H.W., Kanthasamy A.G., Hsu W.H. (2018). Mechanism of intranasal drug delivery directly to the brain. Life Sci..

[25] Samaridou E., Alonso M.J. (2018). Nose-to-brain peptide delivery – The potential of nanotechnology. Bioorg. Med. Chem..

[26] Lochhead J.J., Thorne R.G. (2012). Intranasal delivery of biologics to the central nervous system. Adv. Drug Deliv. Rev..

[27] Mistry A., Stolnik S., Illum L. (2009). Nanoparticles for direct nose-to-brain delivery of drugs. Int. J. Pharm..

[28] Feng Y., He H., Li F., Lu Y., Qi J., Wu W. (2018). An update on the role of nanovehicles in nose-to-brain drug delivery. Drug Discov. Today.

[29] Kozlovskaya L., Abou-Kaoud M., Stepensky D. (2014). Quantitative analysis of drug delivery to the brain via nasal route. J. Control. Release.

[30] Sessa M., Balestrieri M.L., Ferrari G., Servillo L., Castaldo D., D’Onofrio N., Donsì F., Tsao R. (2014). Bioavailability of encapsulated resveratrol into nanoemulsion-based delivery systems. Food Chem..

[31] Savale S., Mahajan H. (2017). Nose to brain: A versatile mode of drug delivery system. Asian J. Biomater. Res..

[32] Pandey M., Choudhury H., Yeun O.C., Yin H.M., Lynn T.W., Tine C.L., Wi N.S., Yen K.C., Phing C.S., Kesharwani P. (2018). Perspectives of Nanoemulsion Strategies in The Improvement of Oral, Parenteral and Transdermal Chemotherapy. Curr. Pharm. Biotechnol..

[33] Sood S., Jain K., Kuppusamy G. (2014). Optimization of curcumin nanoemulsion for intranasal delivery using design of experiment and its toxicity assessment. Colloids Surf. B Biointerfaces.

[34] Choudhury H., Gorain B., Karmakar S., Biswas E., Dey G., Barik R., Mandal M., Pal T.K. (2014). Improvement of cellular uptake, in vitro antitumor activity and sustained release profile with increased bioavailability from a nanoemulsion platform. Int. J. Pharm..

[35] Fernandes C.B., Soni U., Patravale V. (2010). Nano-interventions for neurodegenerative disorders. Pharmacol. Res..

[36] Edmond J. (2001). Essential Polyunsaturated Fatty Acids and the Barrier to the Brain: The Components of a Model for Transport. J. Mol. Neurosci..

[37] Khunt D., Shah B., Misra M. (2017). Role of butter oil in brain targeted delivery of Quetiapine fumarate microemulsion via intranasal route. J. Drug Deliv. Sci. Technol..

[38] Hosny K.M., Banjar Z.M. (2013). The formulation of a nasal nanoemulsion zaleplonin situgel for the treatment of insomnia. Expert Opin. Drug Deliv..

[39] Lin H., Gebhardt M., Bian S., Kwon K.A., Shim C.-K., Chung S.-J., Kim D.-D. (2007). Enhancing effect of surfactants on fexofenadine·HCl transport across the human nasal epithelial cell monolayer. Int. J. Pharm..

[40] Chatterjee B., Gorain B., Mohananaidu K., Sengupta P., Mandal U.K., Choudhury H. (2019). Targeted drug delivery to the brain via intranasal nanoemulsion: Available proof of concept and existing challenges. Int. J. Pharm..

[41] Sood S., Jain K., Gowthamarajan K. (2014). Intranasal therapeutic strategies for management of Alzheimer’s disease. J. Drug Target..

[42] Azeem A., Rizwan M., Ahmad F.J., Iqbal Z., Khar R.K., Aqil M., Talegaonkar S. (2009). Nanoemulsion Components Screening and Selection: A Technical Note. AAPS PharmSciTech.

[43] Morrison E.E., Costanzo R.M. (1992). Morphology of olfactory epithelium in humans and other vertebrates. Microsc. Res. Tech..

[44] Ahmad N., Ahmad R., Naqvi A.A., Alam A., Ashafaq M., Rub R.A., Ahmad F.J. (2017). Intranasal delivery of quercetin-loaded mucoadhesive nanoemulsion for treatment of cerebral ischaemia. Artif. Cells Nanomed. Biotechnol..

[45] Ahmad E., Feng Y., Qi J., Fan W., Ma Y., He H., Xia F., Dong X., Zhao W., Lu Y. (2017). Evidence of nose-to-brain delivery of nanoemulsions: Cargoes but not vehicles. Nanoscale.

[46] Win T., Rajagopal J., Manda U.K., Sengupta P., Chatterjee B. (2017). Incorporation of carbopol to palm olein based analgesic cream: Effect on formulation characteristics. Lat. Am. J. Pharm..

[47] Espinoza L.C., Silva-Abreu M., Clares B., Rodríguez-Lagunas M.J., Halbaut-Bellowa L., Cañas M.-A., Calpena A.C. (2019). Formulation Strategies to Improve Nose-to-Brain Delivery of Donepezil. Pharmaceutics.

[48] Haider F., Khan S., Gaba B., Alam T., Baboota S., Ali J., Ali A. (2018). Optimization of rivastigmine nanoemulsion for enhanced brain delivery: In-vivo and toxicity evaluation. J. Mol. Liq..

[49] Nasr M. (2016). Development of an optimized hyaluronic acid-based lipidic nanoemulsion co-encapsulating two polyphenols for nose to brain delivery. Drug Deliv..

[50] Kumar S., Ali J., Baboota S. (2016). Design Expert^®^ supported optimization and predictive analysis of selegiline nanoemulsion via the olfactory region with enhanced behavioural performance in Parkinson’s disease. Nanotechnology.

[51] Iqbal R., Ahmed S., Jain G.K., Vohora D. (2019). Design and development of letrozole nanoemulsion: A comparative evaluation of brain targeted nanoemulsion with free letrozole against status epilepticus and neurodegeneration in mice. Int. J. Pharm..

[52] Ahmad N., Ahmad R., Alam A., Ahmad F.J., Amir M. (2018). Impact of ultrasonication techniques on the preparation of novel Amiloride-nanoemulsion used for intranasal delivery in the treatment of epilepsy. Artif. Cells Nanomed. Biotechnol..

[53] Abdou E.M., Kandil S.M., El Miniawy H.M. (2017). Brain targeting efficiency of antimigrain drug loaded mucoadhesive intranasal nanoemulsion. Int. J. Pharm..

[54] Bhanushali R.S., Gatne M.M., Gaikwad R.V., Bajaj A.N., Morde M.A. (2009). Nanoemulsion based Intranasal Delivery of Antimigraine Drugs for Nose to Brain Targeting. Indian J. Pharm. Sci..

[55] Yadav S., Gattacceca F., Panicucci R., Amiji M. (2015). Comparative Biodistribution and Pharmacokinetic Analysis of Cyclosporine-A in the Brain upon Intranasal or Intravenous Administration in an Oil-in-Water Nanoemulsion Formulation. Mol. Pharm..

[56] Colombo M., Melchiades G.D.L., Figueiró F., Battastini A.M.O., Teixeira H.F., Koester L.S. (2017). Validation of an HPLC-UV method for analysis of Kaempferol-loaded nanoemulsion and its application to in vitro and in vivo tests. J. Pharm. Biomed. Anal..

[57] Bahadur S., Pathak K. (2012). Buffered nanoemulsion for nose to brain delivery of ziprasidone hydrochloride: Preformulation and pharmacodynamic evaluation. Curr. Drug Deliv..

[58] Boche M., Pokharkar V.B. (2016). Quetiapine Nanoemulsion for Intranasal Drug Delivery: Evaluation of Brain-Targeting Efficiency. AAPS PharmSciTech.

[59] Mehta M., Adem A., Sabbagh M.N. (2012). New Acetylcholinesterase Inhibitors for Alzheimer’s Disease. Int. J. Alzheimer’s Dis..

[60] Arias J.L. (2014). Nanotechnology and Drug Delivery: Volume 1: Nanoplatforms in Drug Delivery.

[61] Surve D.H., Jindal A.B. (2020). Recent advances in long-acting nanoformulations for delivery of antiretroviral drugs. J. Control. Release.

[62] Eqian Z.M., Ke Y. (2014). Huperzine A: Is it an Effective Disease-Modifying Drug for Alzheimer’s Disease?. Front. Aging Neurosci..

[63] Zhang H.-Y. (2012). New insights into huperzine A for the treatment of Alzheimer’s disease. Acta Pharmacol. Sin..

[64] Rascol O., Brooks D.J., Korczyn A.D., De Deyn P.P., Clarke C.E., Lang A.E. (2000). A Five-Year Study of the Incidence of Dyskinesia in Patients with Early Parkinson’s Disease Who Were Treated with Ropinirole or Levodopa. N. Engl. J. Med..

[65] Smith Y., Wichmann T., Factor S., Delong M.R. (2012). Parkinson’s Disease Therapeutics: New Developments and Challenges Since the Introduction of Levodopa. Neuropsychopharmacol..

[66] Jiang Y., Liu C., Zhai W., Zhuang N., Han T., Ding Z. (2019). The Optimization Design of Lactoferrin Loaded HupA Nanoemulsion for Targeted Drug Transport Via Intranasal Route. Int. J. Nanomed..

[67] Singh D., Kapahi H., Rashid M., Prakash A., Majeed A.B.A., Mishra N. (2015). Recent prospective of surface engineered Nanoparticles in the management of Neurodegenerative disorders. Artif. Cells Nanomed. Biotechnol..

[68] Abbott A. (2010). Levodopa: The story so far. Nat. Cell Biol..

[69] Zainol S., Bin Basri H., Bin Basri H., Shamsuddin A.F., Abdul-Gani S.S., Karjiban R.A., Abdul-Malek E. (2012). Formulation Optimization of a Palm-Based Nanoemulsion System Containing Levodopa. Int. J. Mol. Sci..

[70] Pangeni R., Sharma S., Mustafa G., Ali J., Baboota S. (2014). Vitamin E loaded resveratrol nanoemulsion for brain targeting for the treatment of Parkinson’s disease by reducing oxidative stress. Nanotechnology.

[71] Gaba B., Khan T., Haider F., Alam T., Baboota S., Parvez S., Ali J. (2019). Vitamin E Loaded Naringenin Nanoemulsion via Intranasal Delivery for the Management of Oxidative Stress in a 6-OHDA Parkinson’s Disease Model. BioMed Res. Int..

[72] Mustafa G., Baboota S., Ahuja A., Ali J. (2012). Formulation Development of Chitosan Coated Intra Nasal Ropinirole Nanoemulsion for Better Management Option of Parkinson: An In Vitro Ex Vivo Evaluation. Curr. Nanosci..

[73] Sahni J.K., Doggui S., Ali J., Baboota S., Dao L., Ramassamy C. (2011). Neurotherapeutic applications of nanoparticles in Alzheimer’s disease. J. Control. Release.

[74] Kumar M., Bishnoi R.S., Shukla A.K., Jain C.P. (2019). Techniques for Formulation of Nanoemulsion Drug Delivery System: A Review. Prev. Nutr. Food Sci..

[75] Fang J.-Y., Hung C.-F., Chi C.-H., Chen C.-C. (2009). Transdermal permeation of selegiline from hydrogel-membrane drug delivery systems. Int. J. Pharm..

[76] Mustafa G., Ahuja A., Al Rohaimi A.H., Muslim S., Hassan A., Baboota S., Ali J. (2015). Nano-ropinirole for the management of Parkinsonism: Blood–brain pharmacokinetics and carrier localization. Expert Rev. Neurother..

[77] Lipton R.B., Bigal M., Diamond M., Freitag F., Reed M.L., Stewart W.F., on behalf of the AMPP Advisory Group (2007). Migraine prevalence, disease burden, and the need for preventive therapy. Neurology.

[78] Newman L. (2013). Expert commentary. Headache J. Head Face Pain.

[79] Tepper S.J., Cady R., Silberstein S.D., Messina J., Mahmoud R.A., Djupesland P.G., Shin P., Siffert J. (2015). AVP-825 Breath-Powered Intranasal Delivery System Containing 22 mg Sumatriptan Powder vs 100 mg Oral Sumatriptan in the Acute Treatment of Migraines (The COMPASS Study): A Comparative Randomized Clinical Trial Across Multiple Attacks. Headache J. Head Face Pain.

[80] Brodie M.J., Kwan P. (2012). Current position of phenobarbital in epilepsy and its future. Epilepsia.

[81] Kokel D., Peterson R.T. (2008). Chemobehavioural phenomics and behaviour-based psychiatric drug discovery in the zebrafish. Brief. Funct. Genom. Proteom..

[82] Brodie M.J. (2001). Do we need any more new antiepileptic drugs?. Epilepsy Res..

[83] Rivers F., O’Brien T.J., Callaghan R. (2008). Exploring the possible interaction between anti-epilepsy drugs and multidrug efflux pumps; in vitro observations. Eur. J. Pharmacol..

[84] Patsalos P.N. (2005). Properties of Antiepileptic Drugs in the Treatment of Idiopathic Generalized Epilepsies. Epilepsia.

[85] Löscher W., Potschka H. (2005). Drug resistance in brain diseases and the role of drug efflux transporters. Nat. Rev. Neurosci..

[86] Barcia J.A., Gallego J.M. (2009). Intraventricular and intracerebral delivery of anti-epileptic drugs in the kindling model. Neurotherapeutics.

[87] Abdel-Bar H.M., Abdel-Reheem A.Y., Awad G.A.S., Mortada N. (2013). Evaluation of Brain Targeting and Mucosal Integrity of Nasally Administrated Nanostructured Carriers of a CNS Active Drug, Clonazepam. J. Pharm. Pharm. Sci..

[88] Wu X.-Y., Liu J.-S., Shen T., Wang J.-H., Zhou J., Tang X.-H., Xu L., Hong Z. (2014). Enhanced brain delivery of lamotrigine with Pluronic^®^ P123-based nanocarrier. Int. J. Nanomed..

[89] Wilson B., Lavanya Y., Priyadarshini S., Ramasamy M., Jenita J.L. (2014). Albumin nanoparticles for the delivery of gabapentin: Preparation, characterization and pharmacodynamic studies. Int. J. Pharm..

[90] Jain N., Akhter S., Jain G., Khan Z., Khar R., Ahmad F. (2011). Antiepileptic Intranasal Amiloride Loaded Mucoadhesive Nanoemulsion: Development and Safety Assessment. J. Biomed. Nanotechnol..

[91] Kumar M., Pathak K., Misra A. (2009). Formulation and Characterization of Nanoemulsion-Based Drug Delivery System of Risperidone. Drug Dev. Ind. Pharm..

[92] Kumar M., Misra A., Babbar A., Mishra A., Mishra P., Pathak K. (2008). Intranasal nanoemulsion based brain targeting drug delivery system of risperidone. Int. J. Pharm..

[93] Kumar M., Misra A., Pathak K. (2010). Formulation and characterization of nanoemulsion of olanzapine for intranasal delivery. PDA J. Pharm. Sci. Technol..

[94] Kumar M., Misra A., Mishra A.K., Mishra P., Pathak K. (2008). Mucoadhesive nanoemulsion-based intranasal drug delivery system of olanzapine for brain targeting. J. Drug Target..

[95] Parikh V., Tucci V., Galwankar S. (2012). Infections of the nervous system. Int. J. Crit. Illn. Inj. Sci..

[96] DeMarino C., Schwab A., Pleet M., Mathiesen A., Friedman J., El-Hage N., Kashanchi F. (2016). Biodegradable Nanoparticles for Delivery of Therapeutics in CNS Infection. J. Neuroimmune Pharmacol..

[97] Mahajan H., Mahajan M.S., Nerkar P.P., Agrawal A. (2014). Nanoemulsion-based intranasal drug delivery system of saquinavir mesylate for brain targeting. Drug Deliv..

[98] Prabhakar K., Afzal S.M., Surender G., Kishan V. (2013). Tween 80 containing lipid nanoemulsions for delivery of indinavir to brain. Acta Pharm. Sin. B.

[99] Musa S.H., Basri M., Masoumi H.R.F., Karjiban R.A., Malek E.A., Bin Basri H., Shamsuddin A.F. (2013). Formulation optimization of palm kernel oil esters nanoemulsion-loaded with chloramphenicol suitable for meningitis treatment. Colloids Surf. B Biointerfaces.

[100] Scott K.R., Barrett A.M. (2007). Dementia syndromes: Evaluation and treatment. Expert Rev. Neurother..

[101] Birks J., Arrieta J.M.L., López-Arrieta J.M. (2002). Nimodipine for primary degenerative, mixed and vascular dementia. Cochrane Database Syst. Rev..

[102] Pathak R., Dash R.P., Misra M., Nivsarkar M. (2014). Role of mucoadhesive polymers in enhancing delivery of nimodipine microemulsion to brain via intranasal route. Acta Pharm. Sin. B.

[103] (2006). Treatments in depression. Dialog-Clin. Neurosci..

[104] Bahr R., Lopez A., Rey J. (2019). Intranasal Esketamine (SpravatoTM) for Use in Treatment-Resistant Depression in Conjunction with an Oral Antidepressant. Pharm. Ther..

[105] Pandey Y.R., Kumar S., Gupta B.K., Ali J., Baboota S. (2015). Intranasal delivery of paroxetine nanoemulsion via the olfactory region for the management of depression: Formulation, behavioural and biochemical estimation. Nanotechnology.

[106] Mishra D.K., Kumar A., Raj R., Chaturvedi A. (2013). Capmul MCM based nanoemulsion for intranasal delivery of an antidepressant. Bull. Pharm. Res..

[107] Groothuis D.R. (2000). The blood-brain and blood-tumor barriers: A review of strategies for increasing drug delivery. Neuro Oncol..

[108] Dobrovoljac M., Hengartner H., Boltshauser E., Grotzer M. (2002). Delay in the diagnosis of paediatric brain tumours. Eur. J. Nucl. Med. Mol. Imaging.

[109] Basics of Brain Tumors. https://www.hopkinsmedicine.org/health/conditions-and-diseases/basics-of-brain-tumors.

[110] Harter D.H., Wilson T.A., Karajannis M.A. (2014). Glioblastoma multiforme: State of the art and future therapeutics. Surg. Neurol. Int..

[111] Azambuja J.H., Schuh R.S., Michels L.R., Gelsleichter N.E., Beckenkamp L.R., Iser I.C., Lenz G.S., De Oliveira F.H., Venturin G., Greggio S. (2019). Nasal Administration of Cationic Nanoemulsions as CD73-siRNA Delivery System for Glioblastoma Treatment: A New Therapeutical Approach. Mol. Neurobiol..

[112] Colombo M., Figueiró F., Dias A.D.F., Teixeira H.F., Battastini A.M.O., Koester L.S. (2018). Kaempferol-loaded mucoadhesive nanoemulsion for intranasal administration reduces glioma growth in vitro. Int. J. Pharm..

[113] Khan A., Aqil M., Imam S.S., Ahad A., Sultana Y., Ali A., Khan K. (2018). Temozolomide loaded nano lipid based chitosan hydrogel for nose to brain delivery: Characterization, nasal absorption, histopathology and cell line study. Int. J. Biol. Macromol..

[114] Desai A., Vyas T., Amiji M. (2008). Cytotoxicity and Apoptosis Enhancement in Brain Tumor Cells upon Coadministration of Paclitaxel and Ceramide in Nanoemulsion Formulations. J. Pharm. Sci..

[115] Nachiappan K. (2017). Targeted drug delivery system of 5-Fluorouracil with recombinant epidermal growth factor for brain tumor. J. Pharm. Care Health Syst..

[116] Choi K.Y., Liu G., Lee S., Chen X. (2011). Theranostic nanoplatforms for simultaneous cancer imaging and therapy: Current approaches and future perspectives. Nanoscale.

[117] Primo F.L., Rodrigues M.M.A., Simioni A.R., Lacava Z.G.M., Morais P.C., Tedesco A.C. (2008). Photosensitizer-Loaded Magnetic Nanoemulsion for Use in Synergic Photodynamic and Magnetohyperthermia Therapies of Neoplastic Cells. J. Nanosci. Nanotechnol..

[118] De Paula L.B., Primo F.L., Pinto M.R., Morais P.C., Tedesco A.C. (2017). Evaluation of a chloroaluminium phthalocyanine-loaded magnetic nanoemulsion as a drug delivery device to treat glioblastoma using hyperthermia and photodynamic therapy. RSC Adv..

[119] Najlah M., Kadam A., Wan K.-W., Ahmed W., Taylor K.M., Elhissi A. (2016). Novel paclitaxel formulations solubilized by parenteral nutrition nanoemulsions for application against glioma cell lines. Int. J. Pharm..

[120] Pang Z., Feng L., Hua R., Chen J., Gao H., Pan S., Jiang X., Zhang P. (2010). Lactoferrin-Conjugated Biodegradable Polymersome Holding Doxorubicin and Tetrandrine for Chemotherapy of Glioma Rats. Mol. Pharm..

[121] Zhan C., Gu B., Xie C., Li J., Liu Y., Lu W. (2010). Cyclic RGD conjugated poly(ethylene glycol)-co-poly(lactic acid) micelle enhances paclitaxel anti-glioblastoma effect. J. Control. Release.

[122] Gao H., Pang Z., Pan S., Cao S., Yang Z., Chen C., Jiang X. (2012). Anti-glioma effect and safety of docetaxel-loaded nanoemulsion. Arch. Pharmacal Res..

[123] Sarkar S., Raymick J., Imam S.Z. (2016). Neuroprotective and Therapeutic Strategies against Parkinson’s Disease: Recent Perspectives. Int. J. Mol. Sci..

[124] Yadav S., Gandham S.K., Panicucci R., Amiji M. (2016). Intranasal brain delivery of cationic nanoemulsion-encapsulated TNFα siRNA in prevention of experimental neuroinflammation. Nanomed. Nanotechnol. Biol. Med..

[125] Jadidi-Niaragh F., Mirshafiey A. (2010). Histamine and histamine receptors in pathogenesis and treatment of multiple sclerosis. Neuropharmacology.

[126] English C., Aloi J.J. (2015). New FDA-Approved Disease-Modifying Therapies for Multiple Sclerosis. Clin. Ther..

[127] Sarker D.K. (2005). Engineering of Nanoemulsions for Drug Delivery. Curr. Drug Deliv..

[128] Parikh R.H., Patel R.J. (2015). Nanoemulsions for Intranasal Delivery of Riluzole to Improve Brain Bioavailability: Formulation Development and Pharmacokinetic Studies. Curr. Drug Deliv..

[129] Ashafaq M., Khan M.M., Raza S.S., Ahmad A., Khuwaja G., Javed H., Khan A., Islam F., Siddiqui M.S., Safhi M.M. (2012). S-allyl cysteine mitigates oxidative damage and improves neurologic deficit in a rat model of focal cerebral ischemia. Nutr. Res..

[130] Al-Majed A.A., Al-Omar F.A., Nagi M.N. (2006). Neuroprotective effects of thymoquinone against transient forebrain ischemia in the rat hippocampus. Eur. J. Pharmacol..

[131] Ahmad N., Ahmad R., Alam A., Samim M., Iqbal Z., Ahmad F.J. (2016). Quantification and evaluation of thymoquinone loaded mucoadhesive nanoemulsion for treatment of cerebral ischemia. Int. J. Biol. Macromol..

[132] Yero T., Rey J.A. (2008). Tetrabenazine (Xenazine), an FDA-Approved Treatment Option for Huntington’s Disease–Related Chorea. Pharm. Ther..

[133] Wang Z., Xiong G., Tsang W.C., Schätzlein A.G., Uchegbu I.F. (2019). Nose-to-Brain Delivery. J. Pharmacol. Exp. Ther..

[134] Matsuyama T., Morita T., Horikiri Y., Yamahara H., Yoshino H. (2006). Enhancement of nasal absorption of large molecular weight compounds by combination of mucolytic agent and nonionic surfactant. J. Control. Release.

[135] Wen J., Inthavong K., Tu J., Wang S. (2008). Numerical simulations for detailed airflow dynamics in a human nasal cavity. Respir. Physiol. Neurobiol..

[136] Liu Y., Matida E.A., Gu J., Johnson M.R. (2007). Numerical simulation of aerosol deposition in a 3-D human nasal cavity using RANS, RANS/EIM, and LES. J. Aerosol Sci..

[137] Cu Y., Saltzman W.M. (2009). Mathematical modeling of molecular diffusion through mucus. Adv. Drug Deliv. Rev..

